# [1-*tert*-Butyl-3-(pyridin-2-ylmethyl-κ*N*)imidazol-2-yl­idene-κ*C* ^1^]carbonyl­dichlorido(dimethyl sulfoxide-κ*S*)ruthenium(II)

**DOI:** 10.1107/S1600536811042590

**Published:** 2011-10-22

**Authors:** Yong Cheng, Wen-Qian Hua, Ying-Hua Zhou

**Affiliations:** a College of Chemistry and Materials Science, Anhui Normal University, Wuhu 241000, People’s Republic of China

## Abstract

In the title complex, [RuCl_2_(C_13_H_17_N_3_)(C_2_H_6_OS)(CO)], the coordination environment around the Ru atom is slightly distorted octa­hedral. The Cl atoms are mutually *trans* to the dimethyl sulfoxide ligand and the imidazole carbene C atom, respectively. The carbonyl ligand is located *trans* to the pyridine N atom.

## Related literature

For general background to N-heterocyclic carbene (NHC) complexes, see: Hahn *et al.* (2006 ▶); Lee *et al.* (2007 ▶); Mas-Marza *et al.* (2005 ▶); Kaufhold *et al.* (2008 ▶); Araki *et al.* (2008 ▶); Son *et al.* (2004 ▶); Poyatos *et al.* (2006 ▶). For our previous work on Ru–NHC complexes, see: Cheng, Sun *et al.* (2009 ▶); Cheng, Xu *et al.* (2009 ▶).
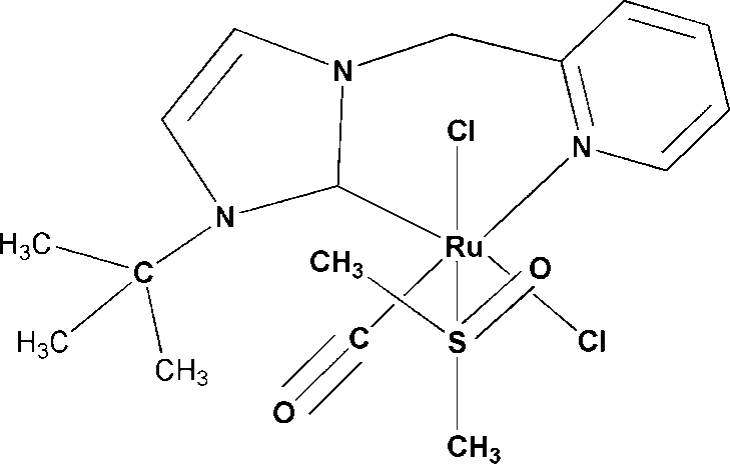


## Experimental

### 

#### Crystal data

[RuCl_2_(C_13_H_17_N_3_)(C_2_H_6_OS)(CO)]
*M* *_r_* = 493.40
Orthorhombic, 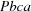

*a* = 14.3297 (14) Å
*b* = 15.7428 (16) Å
*c* = 17.1867 (16) Å
*V* = 3877.1 (7) Å^3^
*Z* = 8
Mo *K*α radiation
μ = 1.21 mm^−1^
*T* = 291 K
0.26 × 0.22 × 0.20 mm

#### Data collection

Bruker SMART APEX CCD diffractometer
Absorption correction: multi-scan (*SADABS*; Sheldrick, 1996 ▶) *T*_min_ = 0.74, *T*_max_ = 0.79
20132 measured reflections
3815 independent reflections
3401 reflections with *I* > 2σ(*I*)
*R*_int_ = 0.044

#### Refinement

*R*[*F* ^2^ > 2σ(*F* ^2^)] = 0.040
*wR*(*F* ^2^) = 0.110
*S* = 1.06
3815 reflections
231 parameters
H-atom parameters constrained
Δρ_max_ = 0.33 e Å^−3^
Δρ_min_ = −1.30 e Å^−3^

### 

Data collection: *SMART* (Bruker, 1997 ▶); cell refinement: *SAINT* (Bruker, 1997 ▶); data reduction: *SAINT*; program(s) used to solve structure: *SHELXS97* (Sheldrick, 2008 ▶); program(s) used to refine structure: *SHELXL97* (Sheldrick, 2008 ▶); molecular graphics: *SHELXTL* (Sheldrick, 2008 ▶); software used to prepare material for publication: *SHELXTL*.

## Supplementary Material

Crystal structure: contains datablock(s) I, global. DOI: 10.1107/S1600536811042590/zb2017sup1.cif

Structure factors: contains datablock(s) I. DOI: 10.1107/S1600536811042590/zb2017Isup2.hkl

Additional supplementary materials:  crystallographic information; 3D view; checkCIF report

## Figures and Tables

**Fig. 1.**
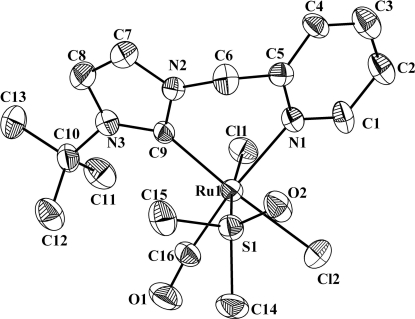
View of the title complex showing 30% probability ellipsoids. Hydrogen atoms are omitted for clarity.[symmetry codes: (i)'-x + 1/2, -y, z + 1/2' (ii)'-x, y + 1/2, -z + 1/2']

## supplementary crystallographic information

### Comment

N-Heterocyclic carbenes (NHCs) complexes have attracted increasing attention as they have been proven to act as efficient homogeneous catalyst (Hahn *et al.* 2006). Pyridine-functionalized bidentate carbene ligands have been frequently used as versatile ancillary ligands in organometallic complexes in recent years (Lee *et al.* 2007). A lot of bidentate pyridinefunctionalized NHC complexes have been prepared, some of which showed catalytic activities in reactions such as hydrosilylation of acetylenes, cyclization of acetylenic carboxylic acids, hydrogen transfer to ketones (Mas-Marza *et al.* 2005). However, few reports have been published on Ru complexes containing bidentate pyridine-functionalized NHC ligands (Kaufhold *et al.* 2008, Araki *et al.* 2008, Son *et al.* 2004, Poyatos *et al.* 2006). We have reported the synthesis and characterization of pyridine functionalized Ru(II)-NHC nitrosyl or carbonyl complexes and their catalytic activity in hydrogen transfer of ketones (Cheng, Sun *et al.*, 2009; Cheng, Xu *et al.*, 2009). Herein, we report a new pyridine functionalized Ru-NHC carbonyl complex with dimethyl sulfoxide.

The structure of the title complex shows that the coordination geometry around the ruthenium atom can be rationalized as a slightly distorted octahedron. Two chloride atoms occupy mutually *trans* to the dimethylsulfoxide and imidazole carbene carbon respectively. The CO group is located *trans* to the pyridine nitrogen (Fig.1).

### Experimental

A mixture of 3-*tert*-butyl-1-picolylimidazolium Bromide (1.0 mmol), silver oxide (1.0 mmol) and CH_2_Cl_2_ (30 ml) was stirred at room temperature for 12 h, and was then filtered through Celite to remove unreacted silver oxide and insoluble residues. [Ru(CO)_2_Cl_2_]_n_ (1.0 mmol) was added to the pale yellow solution, stirred for 12 h at room temperature and then filtered through Celite to remove the silver halide. The products were chromatographed using silica gel. Elution with CH_2_Cl_2_: MeOH (40:1) afforded a pale yellow band that contained the *trans*-[(3-*tert*-butyl-1-picolylimidazol-2-ylidene)biscolorodicarbonylruthenium], Removal of the volatiles under vacuum gave the products as pale yellow powders.

Exposured the saturated dimethyl sulfoxide solution of the *trans*-[(3-*tert*-butyl-1-picolylimidazol-2-ylidene)biscolorodicarbonylruthenium] in air, yellow-rectangle crystals were obtained one month later, which were title complex confirmed by X-ray structure determination. It shows that dimethyl sulfoxide displaced one molecule of CO in previous compound, and the structure converted from *trans* to *cis*.

### Refinement

The structures were solved by direct methods and refined on F^2^ against all reflections by full-matrix least-squares methods with *SHELXTL* program. The hydrogen atoms in the compound were positioned geometrically (C—H = 0.93Å and O—H = 0.83 Å) and refined in the riding-model approximation, with *U*_iso_(H) set to 1.2*U*_eq_(O). All non-hydrogen atoms were refined with anisotropic thermal parameters. The highest peak and deepest hole residual peak in the final difference Fourier map are located at 0.33Å and 1.30 Å, respectively, from atom Ru.

### Crystal data

**Table:**

[RuCl_2_(C_13_H_17_N_3_)(C_2_H_6_OS)(CO)]	*F*(000) = 2000
*M**_r_* = 493.40	*D*_x_ = 1.691 Mg m^−^^3^
Orthorhombic, *P**b**c**a*	Mo *K*α radiation, λ = 0.71073 Å
Hall symbol: -P 2ac 2ab	Cell parameters from 2216 reflections
*a* = 14.3297 (14) Å	θ = 2.3–23.2°
*b* = 15.7428 (16) Å	µ = 1.21 mm^−^^1^
*c* = 17.1867 (16) Å	*T* = 291 K
*V* = 3877.1 (7) Å^3^	Cuboid, yellow
*Z* = 8	0.26 × 0.22 × 0.20 mm

### Data collection

**Table:**

Bruker SMART APEX CCD diffractometer	3815 independent reflections
Radiation source: sealed tube	3401 reflections with *I* > 2σ(*I*)
graphite	*R*_int_ = 0.044
phi and ω scans	θ_max_ = 26.0°, θ_min_ = 2.3°
Absorption correction: multi-scan (*SADABS*; Sheldrick, 1996)	*h* = −16→17
*T*_min_ = 0.74, *T*_max_ = 0.79	*k* = −12→19
20132 measured reflections	*l* = −17→21

### Refinement

**Table:**

Refinement on *F*^2^	Primary atom site location: structure-invariant direct methods
Least-squares matrix: full	Secondary atom site location: difference Fourier map
*R*[*F*^2^ > 2σ(*F*^2^)] = 0.040	Hydrogen site location: inferred from neighbouring sites
*wR*(*F*^2^) = 0.110	H-atom parameters constrained
*S* = 1.06	*w* = 1/[σ^2^(*F*_o_^2^) + (0.07*P*)^2^ + 1.99*P*] where *P* = (*F*_o_^2^ + 2*F*_c_^2^)/3
3815 reflections	(Δ/σ)_max_ < 0.001
231 parameters	Δρ_max_ = 0.33 e Å^−^^3^
0 restraints	Δρ_min_ = −1.30 e Å^−^^3^

### Special details

**Table:**

Experimental. The single crystals was mounted on a glass fibre with silicon grease. Diffraction data were collected on a Bruker *SMART* Apex CCD diffractometer using graphite-monochromated MoKa (l =0.71073 Å) radiation and corrllected for absorption using *SADABS* program.
Geometry. All e.s.d.'s (except the e.s.d. in the dihedral angle between two l.s. planes) are estimated using the full covariance matrix. The cell e.s.d.'s are taken into account individually in the estimation of e.s.d.'s in distances, angles and torsion angles; correlations between e.s.d.'s in cell parameters are only used when they are defined by crystal symmetry. An approximate (isotropic) treatment of cell e.s.d.'s is used for estimating e.s.d.'s involving l.s. planes.
Refinement. Refinement of *F*^2^ against ALL reflections. The weighted *R*-factor *wR* and goodness of fit *S* are based on *F*^2^, conventional *R*-factors *R* are based on *F*, with *F* set to zero for negative *F*^2^. The threshold expression of *F*^2^ > σ(*F*^2^) is used only for calculating *R*-factors(gt) *etc*. and is not relevant to the choice of reflections for refinement. *R*-factors based on *F*^2^ are statistically about twice as large as those based on *F*, and *R*- factors based on ALL data will be even larger.

### Fractional atomic coordinates and isotropic or equivalent isotropic displacement parameters (Å^2^)

**Table:**

	*x*	*y*	*z*	*U*_iso_*/*U*_eq_	
C1	0.7924 (2)	0.1922 (3)	0.4198 (2)	0.0412 (8)	
H1	0.7884	0.1378	0.4404	0.049*	
C2	0.8640 (3)	0.2469 (3)	0.4455 (3)	0.0460 (10)	
H2	0.9069	0.2292	0.4827	0.055*	
C3	0.8685 (3)	0.3279 (3)	0.4137 (3)	0.0504 (10)	
H3	0.9157	0.3649	0.4291	0.060*	
C4	0.8029 (2)	0.3541 (2)	0.3591 (2)	0.0374 (8)	
H4	0.8046	0.4085	0.3380	0.045*	
C5	0.7357 (2)	0.2968 (2)	0.33735 (19)	0.0307 (7)	
C6	0.6614 (3)	0.3243 (2)	0.2791 (2)	0.0389 (8)	
H6A	0.6010	0.3249	0.3047	0.047*	
H6B	0.6748	0.3817	0.2617	0.047*	
C7	0.6571 (3)	0.3016 (3)	0.1371 (2)	0.0487 (10)	
H7	0.6625	0.3586	0.1233	0.058*	
C8	0.6478 (3)	0.2344 (3)	0.0883 (2)	0.0485 (10)	
H8	0.6465	0.2366	0.0343	0.058*	
C9	0.6444 (2)	0.1815 (2)	0.21162 (19)	0.0276 (6)	
C10	0.6203 (3)	0.0776 (3)	0.0934 (2)	0.0403 (8)	
C11	0.6801 (3)	0.0038 (3)	0.1278 (3)	0.0501 (10)	
H11A	0.7451	0.0162	0.1208	0.075*	
H11B	0.6650	−0.0483	0.1016	0.075*	
H11C	0.6670	−0.0020	0.1824	0.075*	
C12	0.5139 (3)	0.0597 (3)	0.1032 (3)	0.0512 (10)	
H12A	0.4976	0.0628	0.1573	0.077*	
H12B	0.4998	0.0040	0.0837	0.077*	
H12C	0.4789	0.1013	0.0746	0.077*	
C13	0.6446 (3)	0.0843 (3)	0.0066 (2)	0.0577 (12)	
H13A	0.6002	0.1206	−0.0188	0.087*	
H13B	0.6425	0.0288	−0.0166	0.087*	
H13C	0.7061	0.1076	0.0008	0.087*	
C14	0.4160 (3)	0.1148 (3)	0.3901 (3)	0.0518 (11)	
H14A	0.4441	0.0782	0.4280	0.078*	
H14B	0.3913	0.0812	0.3482	0.078*	
H14C	0.3664	0.1466	0.4139	0.078*	
C15	0.4341 (3)	0.2282 (3)	0.2722 (3)	0.0524 (10)	
H15A	0.3793	0.2559	0.2920	0.079*	
H15B	0.4158	0.1823	0.2387	0.079*	
H15C	0.4709	0.2682	0.2433	0.079*	
C16	0.5656 (2)	0.0265 (2)	0.2880 (2)	0.0351 (7)	
Cl1	0.78532 (6)	0.05000 (6)	0.29450 (6)	0.0388 (2)	
Cl2	0.63074 (7)	0.05755 (7)	0.44994 (6)	0.0496 (3)	
N1	0.72873 (19)	0.21741 (17)	0.36515 (15)	0.0303 (6)	
N2	0.65692 (19)	0.26818 (19)	0.21148 (17)	0.0325 (6)	
N3	0.6403 (2)	0.1616 (2)	0.13376 (18)	0.0364 (7)	
O1	0.5227 (2)	−0.03364 (19)	0.27652 (19)	0.0556 (8)	
O2	0.51299 (19)	0.25707 (19)	0.40880 (16)	0.0488 (7)	
Ru1	0.636122 (18)	0.119715 (16)	0.318107 (15)	0.02810 (12)	
S1	0.50232 (6)	0.18683 (6)	0.35263 (5)	0.0356 (2)	

### Atomic displacement parameters (Å^2^)

**Table:**

	*U*^11^	*U*^22^	*U*^33^	*U*^12^	*U*^13^	*U*^23^
C1	0.0356 (18)	0.053 (2)	0.0345 (18)	0.0042 (16)	−0.0116 (14)	−0.0115 (16)
C2	0.041 (2)	0.047 (2)	0.050 (2)	−0.0011 (16)	−0.0079 (16)	−0.0176 (19)
C3	0.043 (2)	0.054 (2)	0.054 (3)	−0.0021 (18)	−0.0030 (17)	−0.020 (2)
C4	0.0343 (17)	0.0377 (19)	0.0402 (19)	−0.0088 (15)	0.0031 (14)	−0.0128 (16)
C5	0.0319 (16)	0.0288 (16)	0.0316 (16)	0.0009 (13)	0.0054 (13)	−0.0038 (13)
C6	0.0436 (19)	0.0327 (18)	0.040 (2)	0.0098 (15)	−0.0052 (16)	−0.0053 (15)
C7	0.056 (2)	0.049 (2)	0.041 (2)	−0.0112 (19)	−0.0021 (18)	0.0076 (18)
C8	0.066 (3)	0.044 (2)	0.036 (2)	−0.0120 (19)	−0.0048 (18)	0.0055 (17)
C9	0.0219 (15)	0.0351 (17)	0.0259 (16)	−0.0020 (12)	−0.0013 (11)	−0.0008 (13)
C10	0.0396 (19)	0.051 (2)	0.0300 (18)	−0.0049 (17)	−0.0043 (14)	−0.0075 (16)
C11	0.035 (2)	0.051 (2)	0.064 (3)	0.0041 (17)	−0.0020 (18)	−0.010 (2)
C12	0.034 (2)	0.063 (3)	0.057 (2)	0.0003 (18)	−0.0081 (17)	−0.010 (2)
C13	0.068 (3)	0.075 (3)	0.030 (2)	−0.009 (2)	0.0055 (18)	−0.010 (2)
C14	0.037 (2)	0.063 (3)	0.055 (3)	−0.0163 (18)	0.0153 (18)	−0.011 (2)
C15	0.035 (2)	0.066 (3)	0.057 (2)	0.0095 (18)	−0.0167 (18)	−0.002 (2)
C16	0.0285 (16)	0.0331 (18)	0.0437 (19)	−0.0018 (14)	−0.0075 (14)	0.0018 (15)
Cl1	0.0302 (4)	0.0355 (4)	0.0508 (5)	0.0019 (3)	−0.0024 (3)	−0.0006 (4)
Cl2	0.0601 (6)	0.0520 (6)	0.0366 (5)	−0.0054 (4)	−0.0026 (4)	0.0137 (4)
N1	0.0292 (14)	0.0306 (14)	0.0309 (14)	0.0006 (11)	−0.0028 (10)	−0.0047 (11)
N2	0.0327 (14)	0.0343 (15)	0.0306 (14)	−0.0040 (12)	−0.0035 (11)	0.0037 (12)
N3	0.0383 (16)	0.0379 (16)	0.0331 (16)	−0.0044 (12)	−0.0029 (11)	−0.0039 (13)
O1	0.0553 (17)	0.0423 (16)	0.069 (2)	−0.0183 (14)	−0.0067 (15)	−0.0039 (14)
O2	0.0422 (14)	0.0581 (17)	0.0462 (15)	−0.0040 (13)	0.0044 (12)	−0.0214 (13)
Ru1	0.02643 (17)	0.02881 (18)	0.02905 (18)	−0.00223 (10)	−0.00179 (9)	0.00097 (10)
S1	0.0285 (4)	0.0435 (5)	0.0348 (4)	−0.0005 (3)	0.0018 (3)	−0.0040 (4)

### Geometric parameters (Å, °)

**Table:**

C1—N1	1.368 (4)	C10—C12	1.560 (5)
C1—C2	1.409 (5)	C11—H11A	0.9600
C1—H1	0.9300	C11—H11B	0.9600
C2—C3	1.389 (6)	C11—H11C	0.9600
C2—H2	0.9300	C12—H12A	0.9600
C3—C4	1.391 (6)	C12—H12B	0.9600
C3—H3	0.9300	C12—H12C	0.9600
C4—C5	1.372 (5)	C13—H13A	0.9600
C4—H4	0.9300	C13—H13B	0.9600
C5—N1	1.341 (4)	C13—H13C	0.9600
C5—C6	1.526 (5)	C14—S1	1.797 (4)
C6—N2	1.461 (4)	C14—H14A	0.9600
C6—H6A	0.9700	C14—H14B	0.9600
C6—H6B	0.9700	C14—H14C	0.9600
C7—C8	1.357 (6)	C15—S1	1.815 (4)
C7—N2	1.383 (5)	C15—H15A	0.9600
C7—H7	0.9300	C15—H15B	0.9600
C8—N3	1.391 (5)	C15—H15C	0.9600
C8—H8	0.9300	C16—O1	1.146 (4)
C9—N2	1.376 (4)	C16—Ru1	1.855 (3)
C9—N3	1.376 (5)	Cl1—Ru1	2.4372 (9)
C9—Ru1	2.076 (3)	Cl2—Ru1	2.4692 (10)
C10—N3	1.521 (5)	N1—Ru1	2.186 (3)
C10—C13	1.536 (5)	O2—S1	1.476 (3)
C10—C11	1.560 (6)	Ru1—S1	2.2682 (9)
			
N1—C1—C2	121.6 (4)	C10—C13—H13B	109.5
N1—C1—H1	119.2	H13A—C13—H13B	109.5
C2—C1—H1	119.2	C10—C13—H13C	109.5
C3—C2—C1	118.1 (4)	H13A—C13—H13C	109.5
C3—C2—H2	120.9	H13B—C13—H13C	109.5
C1—C2—H2	120.9	S1—C14—H14A	109.5
C2—C3—C4	120.4 (4)	S1—C14—H14B	109.5
C2—C3—H3	119.8	H14A—C14—H14B	109.5
C4—C3—H3	119.8	S1—C14—H14C	109.5
C5—C4—C3	117.5 (4)	H14A—C14—H14C	109.5
C5—C4—H4	121.2	H14B—C14—H14C	109.5
C3—C4—H4	121.2	S1—C15—H15A	109.5
N1—C5—C4	124.7 (3)	S1—C15—H15B	109.5
N1—C5—C6	116.5 (3)	H15A—C15—H15B	109.5
C4—C5—C6	118.8 (3)	S1—C15—H15C	109.5
N2—C6—C5	112.4 (3)	H15A—C15—H15C	109.5
N2—C6—H6A	109.1	H15B—C15—H15C	109.5
C5—C6—H6A	109.1	O1—C16—Ru1	173.5 (3)
N2—C6—H6B	109.1	C5—N1—C1	117.7 (3)
C5—C6—H6B	109.1	C5—N1—Ru1	124.7 (2)
H6A—C6—H6B	107.9	C1—N1—Ru1	117.1 (2)
C8—C7—N2	105.9 (4)	C9—N2—C7	112.3 (3)
C8—C7—H7	127.1	C9—N2—C6	127.2 (3)
N2—C7—H7	127.1	C7—N2—C6	120.3 (3)
C7—C8—N3	107.7 (4)	C9—N3—C8	110.8 (3)
C7—C8—H8	126.2	C9—N3—C10	130.5 (3)
N3—C8—H8	126.2	C8—N3—C10	118.4 (3)
N2—C9—N3	103.3 (3)	C16—Ru1—C9	98.96 (14)
N2—C9—Ru1	118.3 (2)	C16—Ru1—N1	171.94 (13)
N3—C9—Ru1	138.4 (3)	C9—Ru1—N1	87.79 (11)
N3—C10—C13	109.9 (3)	C16—Ru1—S1	88.94 (11)
N3—C10—C11	111.8 (3)	C9—Ru1—S1	93.48 (9)
C13—C10—C11	107.2 (3)	N1—Ru1—S1	95.09 (7)
N3—C10—C12	107.0 (3)	C16—Ru1—Cl1	94.29 (11)
C13—C10—C12	109.8 (3)	C9—Ru1—Cl1	90.81 (9)
C11—C10—C12	111.2 (3)	N1—Ru1—Cl1	81.13 (7)
C10—C11—H11A	109.5	S1—Ru1—Cl1	174.18 (3)
C10—C11—H11B	109.5	C16—Ru1—Cl2	85.74 (12)
H11A—C11—H11B	109.5	C9—Ru1—Cl2	175.13 (10)
C10—C11—H11C	109.5	N1—Ru1—Cl2	87.62 (8)
H11A—C11—H11C	109.5	S1—Ru1—Cl2	85.31 (4)
H11B—C11—H11C	109.5	Cl1—Ru1—Cl2	90.09 (3)
C10—C12—H12A	109.5	O2—S1—C14	108.05 (19)
C10—C12—H12B	109.5	O2—S1—C15	106.6 (2)
H12A—C12—H12B	109.5	C14—S1—C15	97.4 (2)
C10—C12—H12C	109.5	O2—S1—Ru1	115.63 (11)
H12A—C12—H12C	109.5	C14—S1—Ru1	112.44 (15)
H12B—C12—H12C	109.5	C15—S1—Ru1	115.05 (15)
C10—C13—H13A	109.5		
			
N1—C1—C2—C3	0.0 (6)	C13—C10—N3—C8	19.2 (5)
C1—C2—C3—C4	1.0 (6)	C11—C10—N3—C8	138.1 (4)
C2—C3—C4—C5	−1.1 (6)	C12—C10—N3—C8	−99.9 (4)
C3—C4—C5—N1	0.1 (5)	N2—C9—Ru1—C16	153.9 (2)
C3—C4—C5—C6	178.5 (3)	N3—C9—Ru1—C16	−25.5 (4)
N1—C5—C6—N2	−56.8 (4)	N2—C9—Ru1—N1	−30.6 (2)
C4—C5—C6—N2	124.7 (3)	N3—C9—Ru1—N1	150.1 (3)
N2—C7—C8—N3	1.1 (5)	N2—C9—Ru1—S1	64.4 (2)
C4—C5—N1—C1	0.9 (5)	N3—C9—Ru1—S1	−115.0 (3)
C6—C5—N1—C1	−177.5 (3)	N2—C9—Ru1—Cl1	−111.7 (2)
C4—C5—N1—Ru1	−170.0 (3)	N3—C9—Ru1—Cl1	69.0 (3)
C6—C5—N1—Ru1	11.6 (4)	C5—N1—Ru1—C9	26.9 (3)
C2—C1—N1—C5	−0.9 (5)	C1—N1—Ru1—C9	−144.0 (3)
C2—C1—N1—Ru1	170.7 (3)	C5—N1—Ru1—S1	−66.4 (3)
N3—C9—N2—C7	2.2 (4)	C1—N1—Ru1—S1	122.7 (2)
Ru1—C9—N2—C7	−177.3 (3)	C5—N1—Ru1—Cl1	118.1 (3)
N3—C9—N2—C6	176.7 (3)	C1—N1—Ru1—Cl1	−52.9 (2)
Ru1—C9—N2—C6	−2.8 (4)	C5—N1—Ru1—Cl2	−151.5 (3)
C8—C7—N2—C9	−2.1 (4)	C1—N1—Ru1—Cl2	37.6 (2)
C8—C7—N2—C6	−177.0 (3)	C16—Ru1—S1—O2	158.69 (19)
C5—C6—N2—C9	55.1 (5)	C9—Ru1—S1—O2	−102.40 (17)
C5—C6—N2—C7	−130.8 (4)	N1—Ru1—S1—O2	−14.31 (16)
N2—C9—N3—C8	−1.5 (4)	Cl2—Ru1—S1—O2	72.87 (15)
Ru1—C9—N3—C8	177.9 (3)	C16—Ru1—S1—C14	33.9 (2)
N2—C9—N3—C10	−174.9 (3)	C9—Ru1—S1—C14	132.85 (19)
Ru1—C9—N3—C10	4.5 (6)	N1—Ru1—S1—C14	−139.06 (19)
C7—C8—N3—C9	0.3 (5)	Cl2—Ru1—S1—C14	−51.88 (17)
C7—C8—N3—C10	174.6 (3)	C16—Ru1—S1—C15	−76.3 (2)
C13—C10—N3—C9	−167.8 (3)	C9—Ru1—S1—C15	22.6 (2)
C11—C10—N3—C9	−48.9 (5)	N1—Ru1—S1—C15	110.71 (19)
C12—C10—N3—C9	73.0 (5)	Cl2—Ru1—S1—C15	−162.11 (18)
